# The effects of Spirulina Platensis on anthropometric indices, appetite, lipid profile and serum vascular endothelial growth factor (VEGF) in obese individuals: a randomized double blinded placebo controlled trial

**DOI:** 10.1186/s12906-017-1670-y

**Published:** 2017-04-21

**Authors:** Reihaneh Zeinalian, Mahdieh Abbasalizad Farhangi, Atefeh Shariat, Maryam Saghafi-Asl

**Affiliations:** 1 Nutrition Research Center, Department of Nutrition in Community, Faculty of Nutrition, Tabriz University of Medical Sciences, Tabriz, Iran; 2 Drug Applied Research Center, Nutrition Research Center, Department of Nutrition in Community, Faculty of Nutrition, Tabriz University of Medical Sciences, Tabriz, Iran; 3 Student Research Committee, Department of Nutrition in Community, Faculty of Nutrition, Tabriz University of Medical Sciences, Tabriz, Iran

**Keywords:** Spirulina Platensis, Vascular endothelial growth factor (VEGF), Lipid profile, Appetite

## Abstract

**Background:**

In recent years, a great attention has been focused on Spirulina platensis as a source of potential valuable nutrients for prevention and treatment of chronic diseases. The objectives of the current study were to determine the effects of Spirulina platensis on anthropometric parameters, serum lipids, appetite and serum Vascular Endothelial Growth Factor (VEGF) in obese individuals.

**Methods:**

In the current study sixty four obese individuals aged 20–50 years were enrolled and randomly allocated into two groups of intervention and placebo. Intervention group (*n* = 29) received each 500 mg of the Spirulina platensis a twice-daily dosage while the control group (*n* = 27) received two pills daily starch for 12 weeks. Anthropometric parameters and serum VEGF and lipid profile were measured in fasting blood samples at the beginning and end of the study period. Dietary intakes were assessed by a 24-h recall method and appetite was measured using standard visual analogue scale (VAS).

**Results:**

Body weight and body mass index (BMI) were decreased in intervention and placebo treated groups although the mean reduction in Spirulina platensis-treated group was significantly higher (*P* < 0.05). Serum total cholesterol (TC) significantly reduced in intervention group (*P* < 0.05). Also, treatment with Spirulina platensis significantly reduced appetite (*P* = 0.008). Mean serum VEGF, low density lipoprotein-cholesterol, and triglycerides did not change significantly after intervention. Serum high density lipoprotein-cholesterol concentrations (HDL-c) significantly increased in both groups while no difference in mean difference of this change has been observed.

**Conclusion:**

Spirulina supplementation at a dose of 1 g/d for 12 weeks is effective in modulating body weight and appetite and partly modifies serum lipids. This can further confirm the efficacy of this herbal supplement in control and prevention of obesity and obesity- related disorders.

**Trial registration:**

Iranian registry of clinical trials (IRCT registration number: IRCT2015071219082N7; Date registered: September 12, 2015).

## Background

Obesity is one of the main health problems throughout the world and its prevalence is increasing rapidly [1]. Obesity is associated with numerous health related comorbidities like cardiovascular events, diabetes, metabolic syndrome, hypertension and even some types of cancers [2]. According to the recent report by the World Health Organization (WHO), in 2014 more than 600 million adults were obese. Overweight and obesity are the fifth leading risk for global deaths [3]. At least 2.8 million adults die each year as a result of obesity [2, 4].

Diet and physical activity play an important role in regulating weight. In recent years use of herbs for weight loss has been dramatically increased, because of fewer side effects of herbal remedies [1, 5]. Recent studies reported that long-term obesity causes the release of some factors that can promote proliferative disorders in the gland, culminating in diffuse hyperplasia [6]. As observed in human adipose tissue, one mechanism for an increased inflammatory response may arise through activation of the innate immune system [7]. Adipose tissue contains abundant endothelial cells that could secrete angiogenic factors, such as vascular endothelial growth factor (VEGF) [8]. VEGF is an important angiogenic factor implicated in normal and pathological vessel formation [9], which is an important biomarker in obesity and obesity-related cancer progression [10]. Increased serum VEGF concentrations due to visceral fat accumulation could also influence vascular endothelial function [9].

On the other hand, many supplements have been manufactured in order to decrease obesity and its negative consequences on health. Spirulina (Arthrospira platensis) is a filamentous cyanobacterium used as a food supplement [11]. WHO projects that Spirulina will become one of the most curative and prophylactic components of nutrition in the twenty-first century [12]. It is not expensive and some researchers have even named it a “super-food” [13]. In recent years, great attention has been paid to Spirulina platensis as a source of a potential treatment for many diseases [14–17]. Very limited studies evaluated the anti-obesity effects of Spirulina platensis. In one animal model by Balasubramanian et al. [18], treatment with Spirulina platensis significantly reduced weight in high fat diet induced obese rats. To our review of literature, no human study was available examining the effects of Spirulina platensis on obesity and its metabolic consequences. We hypothesized that Spirulina platensis treatment is capable of modulating serum lipids, body weight, appetite control and reducing serum VEGF concentrations. Therefore, the objectives of the current study was to determine the effects of a twice-daily dosage (each 500 mg) of the Spirulina platensis supplement for 12 weeks on anthropometric features, lipid profile, appetite and serum VEGF concentrations in obese individuals.

## Methods

### Study design

**Fig. 1:**
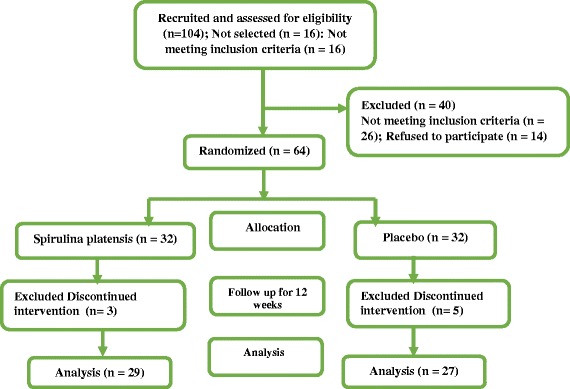
Study flowchart

In the current randomized double blind placebo-controlled trial sixty four obese individuals aged 20–50 years were enrolled (Fig. 1)**.** Inclusion criteria included: BMI ≥ 30 kg/m^2^ and age of 20–50 years. Exclusion criteria included: any history of kidney diseases, atherosclerosis, cancer, acute infections, recent surgery, use of hormonal medications, anti-depressive medications, antibiotics, anti-diuretics, glucocorticoids and using vitamin or mineral supplements in the past three months, pregnancy, lactation, menopause and being on weight losing diets in the past three months. The protocol the current study was approved by the ethics committee of Tabriz University of Medical Sciences (Registration number: 1394.461). Individuals were informed about the study aims and a time of 10 days prior participation in the trial was given to them to announce their participation in the study. Written informed consent was obtained from all participants.

### Intervention

Participants were allocated to treatment groups by simple randomization. They were divided into two groups, matched by age and gender. In the intervention group (*n* = 29), a twice-daily dosage (each 500 mg) of the Spirulina platensis supplement (Far East Microalgae Ind. Co., LTD, Femico, Taiwan) was administered. The contents of Spirulina platensis supplement are presented in Table 1. The control group (*n* = 27) received two pills daily, which contained one gram starch without chlorophyll as a placebo in similar-looking and shape with spirulina platensis pills. The duration of study was 12 weeks. Participants were followed by phone contacts every 7 days, in order to verify that they had taken the pills. Participants were also asked some questions in order to ascertain whether or not they kept to their usual diet. They were requested not to take any mineral or vitamin supplements and also not to change their normal diet or their usual physical activity.

**Table 1:** Contents of the Spirulina platensis supplement^a^

Spirulina platensis ingredients	Content (per gram)
Total protein	550–670 mg
Total fat	60–80 mg
Total fiber	20–60 mg
chlorophyll	15 mg
Total ash	60–80 mg
carbohydrate	120–200 mg
Moisture	40–60 mg
Beta Carotene	2.58 mg
Vitamin E	0.066 mg
Niacin	0,16 mg
Phosphorus	9.14 mg
Sodium	1.86 mg
Calcium	1.71 mg
Magnesium	2.6 mg
Potassium	17.7 mg
Iron	0.75 mg
Zinc	0.05 mg
Vitamin B1	48 mg
Vitamin B2	42 mg
Vitamin B6	7 mg
Vitamin B12	2.3 mg
Biotin	0.25 mg
Folic Acid	0.61 mg

^a^Provided by Far East Microalgae Ind. Co., LTD, Femico, Taiwan

### Anthropometric and dietary assessments

Body weight was measured with a calibrated digital scale and height was measured with a stadiometer. BMI was calculated as weight (kg) divided by height (cm) squared. During the anthropometric measurements, subjects wore lightweight clothing and no shoes. Weight was measured to the nearest 0.1 kg and height was measured to the nearest 0.1 cm. Dietary intakes were assessed by a 24-h food-recall for three days (two on working days and one on a weekend) at the beginning and at end of the study, in order to determine if there was a major modification in the individuals’ usual dietary intake that would exclude them from analysis. Dietary intakes were analyzed by Nutritionist IV software.

### Measurements of appetite

Appetite was measured with visual analogue scale (VAS). VAS is a standard tool used to measure subjective appetite completed before and after every meal. VAS includes 10 items about feeling of hunger, fullness, appetite, satiety, thirst, prospective food consumption and desire to eat. Moreover, VAS is most often composed of lines (of varying length) with words anchored at each end, describing the extremes (that is, “I have never been more hungry”/“I am not hungry at all”). Subjects were asked to make a mark across the line corresponding to their feelings. Quantification of the measurement was done by measuring the distance from the left end of the line to the mark [19]. These scales were administered at intervals of 10–30 min during studies. Subjects were familiarized with these scales prior to the commencement of the study.

### Physical activity level

The questionnaire with nine different metabolic equivalent (MET) scales ranging from sleep/rest (0.9 METs) to high-intensity physical activates (>6 METs) was used to determine physical activity levels. The MET values were multiplied by the time spent at that particular level for each activity level. At each level, the MET-time was added to obtain a total over 24 h MET-time, demonstrating the physical activity level on an average day. Physical activities of different intensities were rated to three categories, termed as sedentary (<3 METs), moderate (3–6 METs) and vigorous (>6 METs) [20].

### Biochemical assays

Fasting blood samples were taken at the beginning and end of the twelve weeks of intervention. The serum and plasma samples were separated by centrifugation at 2500 rpm for 10 min (Beckman Avanti J-25; Beckman Coulter, Brea, CA, USA) at room temperature. The serum samples were stored at −70 °C immediately after centrifugation. Serum VEGF was measured with the Human VEGF ELISA Kit (Hangzhou East biopharm Co., LTD, USA). The intra-assay and inter-assay coefficient of variation (CV) for VEGF assessment were <15%. Serum lipids were assessed by Abbott ALCYON™ 300 auto analyzer using commercial ELISA kits (Pars-Azmoon, Tehran, Iran). All of the biochemical assays were performed by a trained lab assistant who was blinded to group assignments. Serum low density lipoprotein cholesterol- concentrations (LDL-c) was calculated by Friedewald formula [21].

### Statistics

Statistical analysis was performed with the Statistical Package for Social Sciences (SPSS) version 21of software package (SPSS Inc., Chicago, IL, USA). The Kolmogorov-Smirnov test was used to assess the normality of the data. Quantitative data were presented as mean ± standard deviation (SD), and qualitative data were demonstrated as frequency and percent. A Chi-square test was performed to determine differences at the baseline in frequencies of categorized variables between the groups. Between the groups, comparisons of continuous variables were performed by independent sample t-tests. The paired *t*-test was used to analyze intra-group change of all measured parameters between the baseline and end of the intervention period. Analysis of covariance (ANCOVA) was used to identify any differences between the two treatment groups after intervention, adjusting for the confounding effects of baseline concentrations of parameter, age, gender and physical activity level in three models. *P* value of less than 0.05 was considered statistically significant. Sample size calculation was performed based on 80% power and an a-error of 5% to detect treatment effect of Spirulina platensis on serum LDL-c [22]; a total of 28 individuals were calculated in each group. Allowing for 15% drop-out over 12 weeks of intervention, the total sample size required for the study is 64 individuals with 32 individuals in each group.

## Results

### General characteristics of trial and drop-outs

The flowchart of the study has been shown in Fig. 1. In Spirulina platensis group three participants and in control group five participants declined to continue the trial. Finally 56 individuals completed the study. In the current study no side effects of treatment were observed.

### Demographic parameters, physical activity level and nutrients intake

Demographic parameters and physical activity levels of the subjects are reported in Table 2. No significant difference in gender distribution and physical activity level was observed at the beginning and end of treatment. The intake of nutrients during the study was constant and was comparable between the groups (Table 3).

**Table 2:** General characteristics of study participants

Variable		Spirulina platensis	Placebo	P†
Gender [*n* (%)]	Male	5 (17.2)	4 (14.8)	0.81
	female	24 (82.8)	23 (85.2)	
Age (y)		34.75 ± 8.04	33.92 ± 8.57	0.71
Physical activity				
Before	*Low*	16 (55.2)	12 (44.4)	0.80
	*Moderate*	12 (41.4)	14 (51.9)	
	*High*	1 (3.4)	1 (3.7)	
After	*Low*	11 (37.9)	6 (22.2)	0.91
	*Moderate*	17 (58.6)	20 (74.1)	
	*High*	1 (3.4)	1 (3.7)	
P‡		*0.03*	0.12	

**†** *P* values for independent *t*-test**, ‡** *P* values for paired *t*-test. The statistically significant values are presented as italic digits

**Table 3:** Dietary intake of energy, macro and micronutrients in study groups before and after treatment

Variable		Spirulina platensis (Mean ± SD)	Placebo (Mean ± SD)	P†
Energy (kcal)	*Before* *After* *P* ^*‡*^	2289.51 ± 436.48 2289.96 ± 361.78 0.99	2305.71 ± 298.84 2266.47 ± 347.24 0.40	0.418
Protein (gr)	*Before* *After* *P* ^*‡*^	103.8 ± 25.58 110.5 ± 26.19 0.06	110.24 ± 31.9 113.14 ± 24.8 0.62	0.70
Carbohydrate (gr)	*Before* *After* *P* ^*‡*^	303.67 ± 105.27 299.34 ± 81.63 0.73	302.3 ± 79.58 304.13 ± 61.48 0.9	0.80
Fat (gr)	*Before* *After* *P* ^*‡*^	73.67 ± 28.54 70.54 ± 20.03 0.4	67.49 ± 24.61 68.77 ± 21.48 0.79	0.75
Protein (%)	*Before* *After* *P* ^*‡*^	18.20 ± 4.10 18.72 ± 5.58 0.5	19.59 ± 5.92 19.25 ± 3.85 0.79	0.68
Carbohydrate (%)	*Before* *After* *P* ^*‡*^	50.37 ± 12.37 51.96 ± 8.55 0.54	54.48 ± 9.54 53.07 ± 6.75 0.54	0.59
Fat (%)	*Before* *After* *P* ^*‡*^	27.95 ± 8.57 29.1 ± 6.78 0.36	25.92 ± 7.42 28.92 ± 10.66 0.18	0.94
Vitamin E(mg)	*Before* *After* *P* ^*‡*^	9.75 ± 8.34 11.02 ± 11.58 0.316	8.00 ± 9.2 10.23 ± 10.27 0.21	0.14
Vitamin C(mg)	*Before* *After* *P* ^*‡*^	81.23 ± 39.94 93.05 ± 33.43 0.12	74.36 ± 43.35 67.92 ± 33.31 0.41	*0.007*
Selenium(mg)	*Before* *After* *P* ^*‡*^	0.1 ± 0.05 0.14 ± 0.18 0.25	0.12 ± 0.17 0.08 ± 0.04 0.33	0.1

‡ *P* values for paired *t*-test;†*P* values based on ANCOVA after adjustment for age, gender, physical activity, and variable’s baseline value. The statistically significant values are presented as italic digits

### Change in anthropometric and biochemical parameters

BMI had significantly decreased in both groups and its mean reduction in Spirulina platensis-treated group was significantly higher compared with placebo-treated group. Serum VEGF had not statistically changed (Table 4).There were no significant differences on TG and LDL in the intervention or control group. HDL significantly increased in both intervention and control group (*P* < 0.05), although mean differences of change in serum HDL-c between groups were not significant (*P* = 0.514). Total cholesterol had significantly decreased in subjects who received Spirulina (*P* = 0.002), whereas there was no significant difference in the control group (*P* = 0.086). Treatment with Spirulina platensis also significantly reduced appetite (*P* = 0.008). No change in serum VEGF and other serum lipids were observed. Adjustment for the confounding effects of variables baseline concentrations, age, gender and physical activity was performed by ANCOVA test in three models (Table 5); in this model, when variables’ baseline value, gender and age were included in the model as confounding variables, weight, BMI and appetite showed significant difference between treatment groups; while when physical activity was also inserted into ANCOVA model, weight lost its significant level, but BMI and appetite remained significant.

**Table 4:** Anthropometric and metabolic parameters in study groups before and after treatment

Variable		Treatment Groups				*P* ^*‡*^
		Spirulina platensis	*Percent change*	Placebo	*Percent change*	
Weight(kg)	*Before*	89.62 ± 11.54	1.79	87.66 ± 13.64	0.71	0.007
	*After*	88.01 ± 11.42		87.03 ± 13.70		
	*P†*	*<0.001*		**0.09**		
WC(cm)	*Before*	98.89 ± 8.48	1.40	101.07 ± 9.56	0.66	0.059
	*After*	97.50 ± 8.39	100.40 ± 9.77			
	*P†*	*0.00*	*0.003*			
BMI (kg/m2)	*Before*	33.35 ± 2.81	−1.90	32.71 ± 3.18	−0.73	0.005
	*After*	32.71 ± 2.80		32.47 ± 3.24		
	*P†*	*<0.001*		*0.01*		
VEGF(ng/l)	Before	3.22 ± 0.37	0.14	3.20 ± 0.28	**0.25**	0.580
	after	3.22 ± 0.37		3.21 ± 0.29		
	*P†*	**0.775**		**0.735**		
TG(mg/dl)	*Before*	144.13 ± 57.57	−3.84	156.14 ± 79.01	−7.31	0.486
	*after*	136.65 ± 60.80		140.88 ± 72.73		
	*P†*	**0.365**		*0.052*		
LDL(mg/dl)	*Before*	116.27 ± 34.79	−2.06	119.90 ± 21.69	−2.05	0.725
	*after*	115.42 ± 28.61		116.68 ± 21.31		
	*P†*	**0.886**		**0.196**		
HDL(mg/dl)	*Before*	36.55 ± 10.21	1.73	34.88 ± 11.56	4.36	0.385
	*after*	38.75 ± 8.84		38.37 ± 9.44		
	*P†*	*0.05*		*0.001*		
TC(mg/dl)	Before	190.48 ± 35.25	−4.67	187.25 ± 27.10	−2.18	0.123
	after	180.10 ± 31.13		183.03 ± 28.07		
	*P†*	*0.002*		**0.09**		
Appetite	Before	211.03 ± 31.68	−4.16	191.29 ± 33.95	2.44	*0.001*
	after	202.06 ± 34.13		195 ± 33.25		
	*P†*	*0.008*		**0.064**		

*WC* waist circumference, *BMI* body mass index, *VEGF* vascular endothelial growth factor, *TG* triglyceride, *LDL* low density lipoprotein, *HDL* high density lipoprotein, *TC* total cholesterol

†*P* values for paired *t*-test; ‡ *P* values obtained by the comparison of mean differences by independent sample *t-*test. The statistically significant values are presented as italic digits

**Table 5:** The comparison of studied variables between study groups after adjusting for possible confounders

Variable	Model 1	Model 2	Model 3
Weight (kg)	*0.026*	*0.057*	0.106
WC (cm)	0.168	0.255	0.403
BMI (kg/m^2^)	*0.017*	*0.038*	*0.038*
VEGF(ng/l)	0.820	0.918	0.876
TG (mg/dl)	0.394	0.513	0.533
LDL(mg/dl)	0.445	0.659	0.735
HDL(mg/dl)	0.636	0.755	0.441
TC(mg/dl)	0.080	0.170	0.180
Appetite	*0.004*	*0.012*	*0.019*

*WC* waist circumference, *BMI* body mass index, *VEGF* vascular endothelial growth factor, *TG* triglyceride, *LDL* low density lipoprotein, *HDL* high density lipoprotein, *TC* total cholesterol, Model 1, *P* values obtained by ANCOVA after adjusting for the variables baseline value, and gender, Model 2, *P* values obtained by ANCOVA after adjusting for the variables baseline value, gender and age, Model 3, *P* values obtained by ANCOVA after adjusting for the variables baseline value, gender, age and physical activity. The statistically significant values are presented as italic digits

## Discussion

Spirulina is a blue-green alga known as nutritious food with a high content of proteins, vitamins, minerals and antioxidants [23–25]. Numerous studies had reported health benefits of Spirulina in some diseases such as anemia, diabetes, arthritis, cancer and cardiovascular disorders [24]. On the other hand, it has been claimed that Spirulina has potential positive effects in therapeutic management of chronic metabolic and non-metabolic disorders [26].

The current study for the first time evaluated the beneficial effects of Spirulina platensis in obesity and its metabolic consequences in obese individuals. We demonstrated anti-obesity effects of this herbal medicine and its therapeutic effects on serum TC and appetite control.

In the present study Spirulina platensis did not have significant effects on serum VEGF in healthy obese individuals in comparison to the control group. Although in a previous animal study, treatment with Spirulina (200 and 400 mg/kg) reduced serum VEGF in alkali burn-induced corneal inflammation and neovascularization among rats [17]. Epidemiological studies suggested that obesity is associated with increased levels of adipose tissue derived growth factors like VEGF [27]. Higher plasma levels of VEGF in obese human have been observed by some studies [9, 28]. It has also been reported that overweight and obese young adult women had a higher plasma level of unbound-VEGF than lean young adult women [10]. However, some other studies failed to confirm these findings [29]. The discrepancies in findings of different studies could be attributed to difference in the obesity level of participants, the study sample size or the study design.

In the current study we observed a significant reduction in serum TC although other lipids did not changed. Similarly, previous studies reported that 12 weeks of Spirulina supplementation at a dose of 1 g/day has powerful hypolipidemic effects, especially on the serum triglyceride concentration in dyslipidemic Cretan outpatients [11]. Also, four g/day of Spirulina on Egyptian patients with hyperlipidemia was effective [30]. In another study, Spirulina (4.2 g/day) was added for eight weeks to the diet of 30 Japanese males with high cholesterol, mild hypertension, and hyperlipidemia. It lowered total cholesterol, triglycerides and increased HDL [15]. Possibly Spirulina platensis exerts its hypolipidemic effects via increase in lipoprotein lipase and hepatic triglyceride lipase activity [31]. The hypolipidemic activities of Spirulina platensis has been attributed to its active ingredient Phycocyanin [32]; Phycocyanin is a water soluble protein and enriched in Spirulina. Ingestion of phycocyanin preparation resulted in a significant decrease in serum total cholesterol and atherogenic index whereas serum HDL cholesterol was concurrently increased. Moreover, fecal excretion of cholesterol and bile acid was also increased. It was proposed that decreases in intestinal cholesterol and bile acid absorption following Spirulina platensis feeding may represent a mechanism for the hypocholesterolemic action of its major ingredient [32].

In this study, we mentioned a significant decrease in appetite and consequently weight and BMI in intervention group. The weight reducing effects of Spirulina platensis could be attributed to its appetite reducing effect as observed in the current study. Since gender, age and physical activity could affect the results as confounding parameters, we adjusted the analysis for these parameters by ANCOVA test. We used three models which included gender (model 1), gender and age (model 2), gender, age and physical activity (model 3) to reach the goal. The results approved that appetite, weight and BMI reduction was not under the influence of these confounders except “weight” for model 3. A recent study demonstrated that three months of taking of 2 g/day Spirulina improves BMI and weight as well as blood pressure in overweight patients with hypertension without evidence of cardiovascular disease [33]. In the mentioned study, gender and age were considered as parameters with confounding effects and the results were adjusted for these confounders.

## Conclusion

In conclusion, the findings of the present study demonstrated that Spirulina platensis supplementation at a dose of 1 g daily was effective in weight regulation, serum total cholesterol and appetite reduction. Several limitations of the current study should also be mentioned; low sample size and short period of treatment could be involved in minor discrepancy of our findings with our hypothesis of change in serum VEGF or other lipids. Although the current study was the first one evaluating the healthful beneficial effects of Spirulina platensis in obesity. Further studies with higher dose, sample sized and study duration are warranted.

## Abbreviations

BMI: Body mass index
HDL: High density lipoprotein concentrations
LDL: Low density lipoprotein concentrations
TC: Total cholesterol
VAS: Visual analogue scale
VEGF: Vascular endothelial growth factor
VLDL: Very low density lipoprotein concentrations
WHO: World Health Organization
